# Bird expertise does not increase motion sensitivity to bird flight motion

**DOI:** 10.1167/jov.21.5.5

**Published:** 2021-05-05

**Authors:** Simen Hagen, Quoc C. Vuong, Michael D. Chin, Lisa S. Scott, Tim Curran, James W. Tanaka

**Affiliations:** 1 Department of Psychology, University of Victoria, Victoria, BC, Canada; 2 Donders Institute for Brain, Cognition and Behaviour, Radboud University, Nijmegen, The Netherlands; 3 Biosciences Institute, Newcastle University, Newcastle upon Tyne, United Kingdom; 4 Department of Psychology, University of Victoria, Victoria, BC, Canada; 5 Department of Psychology, University of Florida, Gainesville, FL, USA; 6 Department of Psychology and Neuroscience, University of Colorado Boulder, Boulder, CO, USA; 7 Department of Psychology, University of Victoria, Victoria, BC, Canada

**Keywords:** biological motion recognition, perceptual expertise, real-world expertise, object recognition, motion

## Abstract

While motion information is important for the early stages of vision, it also contributes to later stages of object recognition. For example, human observers can detect the presence of a human, judge its actions, and judge its gender and identity simply based on motion cues conveyed in a point-light display. Here we examined whether object expertise enhances the observer's sensitivity to its characteristic movement. Bird experts and novices were shown point-light displays of upright and inverted birds in flight, or upright and inverted human walkers, and asked to discriminate them from spatially scrambled point-light displays of the same stimuli. While the spatially scrambled stimuli retained the local motion of each dot of the moving objects, it disrupted the global percept of the object in motion. To estimate a detection threshold in each object domain, we systematically varied the number of noise dots in which the stimuli were embedded using an adaptive staircase approach. Contrary to our predictions, the experts did not show disproportionately higher sensitivity to bird motion, and both groups showed no inversion cost. However, consistent with previous work showing a robust inversion effect for human motion, both groups were more sensitive to upright human walkers than their inverted counterparts. Thus, the result suggests that real-world experience in the bird domain has little to no influence on the sensitivity to bird motion and that birds do not show the typical inversion effect seen with humans and other terrestrial movement.

## Introduction

In the human visual system, specific processes are dedicated to the detection and encoding of movement in the natural world. For example, in early stages of vision, movement attracts attention, allowing for rapid detection of moving entities. In later stages of vision, the characteristic movements associated with natural objects (e.g., humans and animals) signal behavioral relevance. An open question is whether extensive perceptual experience to objects from natural category domains enhances the observer's sensitivity to its characteristic movement. In the perceptual expertise literature, there is substantial evidence that real-world experts recognize objects from their domain of expertise (e.g., birds, dogs, cars, humans) more quickly and accurately than novices (e.g., Bukach et al., 2006; Hagen et al., 2014, 2016; Tanaka & Taylor, 1991). Subsequent research has shown that this recognition advantage may be due in part to the findings that experts utilize color (e.g., Hagen et al., 2014) and spatial-frequency information (e.g., Hagen et al., 2016) in their expertise domain more efficiently than novices. Thus, similar to these other visual dimensions, motion sensitivity could potentially increase as a result of extensive real-world experience (e.g., birdwatching). There is strong evidence that this is the case for human motion, another natural category for which observers can be considered experts. However, the hypothesis that expertise can increase motion sensitivity has not been tested beyond the context of human motion. In the current study, we therefore directly compared experienced birdwatchers’ and bird novices’ sensitivity to the articulatory movements during flight or locomotion of birds and humans, respectively, in the absence of other visual dimensions such as color or spatial frequency.

As noted, adult observers can be considered human experts. They have a lifetime of experience watching other people, and these experiences may tune perceptual and neural mechanisms for processing human-specific movements. Moreover, adult humans interact with others in daily social activities and can perform similar body movements (Johnson & Shiffrar, 2013). The seminal psychophysical work by Johansson (1973) showed that observers recognized human movement based on stimuli in which form and color were abolished. Specifically, light bulbs were placed on strategic joints of a human before filming different actions in the dark, producing so-called point-light (PL) videos. Human observers presented with the PL videos instantly recognized the presence of a human and their movements. Moreover, still frames of the videos were not sufficient for recognition, indicating that the crucial cues were contained in the spatiotemporal characteristics of the dots. Studies have shown that a variety of complex human motion patterns can be extracted from PL displays, including a variety of full-body actions (Dittrich, 1993), arm movements (Pollick, Paterson, Bruderlin, & Sanford, 2001), and facial expressions (Bassili, 1978). Human observers can even detect the subtle motion cues associated with someone's identity (Cutting & Kozlowski, 1977), the gender of the walker (Kozlowski & Cutting, 1977; Troje, 2002), and emotional states (Dittrich, Troscianko, Lea, & Morgan, 1996; Pollick et al., 2001). There is evidence that there are mechanisms that incidentally process human articulations in PL displays in a “bottom-up” manner (Cavanagh et al., 2001; Thornton & Vuong, 2004; Troje & Westhoff, 2006) and that these mechanisms can generalize to human articulations in natural scenes (Mayer, Vuong, & Thornton, 2015, 2017). Notably, the sensitivity for articulatory motion is tuned for the upright orientation, most likely because humans and other terrestrial animals are encountered upright (e.g., Cutting et al., 1988; Grossman, Blake, & Kim, 2004; Hiris, Humphrey, & Stout, 2005). Presenting PL displays of inverted human motion can often impair detection and recognition, leading to a so-called inversion effect (Ikeda, Blake, & Watanabe, 2005; Pavlova & Sokolov, 2000; Sumi, 1984). The upright advantage could be supported by a specialized process that is either less optimally, or not recruited at all, by inverted humans, and this tuning could be due to perceptual learning during an individual's lifetime or due to a specialized process forged by evolutionary pressures (or a combination of the two).

Further evidence suggests that motion-specific processes are susceptible to perceptual learning in an individual's lifetime. For example, human adults acquire knowledge of motion cues as evidenced by their ability to identify humans by their gait (Cutting & Kozlowski, 1977) or by recognizing culturally relevant actions (e.g., chopping wood, baseball batting; Pica et al., 2011). This experience can lead to differential neural sensitivity to human compared to animal motion due to constant engagement in person perception (Kaiser et al., 2012; Pinto & Shiffrar, 2009). Experimental work also showed that observers are able to learn, through laboratory training, PL displays of novel human biological motion, created by linearly combining different prototypical biological movements across space and time (marching, running, or aerobics) and novel “animal-like” terrestrial motion (e.g., walking; Jastorff, Kourtzi, & Giese, 2002, 2006; Pyles et al., 2007). Moreover, perceptual learning can improve sensitivity to already stored human motion templates. For example, observers trained to detect PL displays of different human actions embedded in random noise dots improve their sensitivity to the trained actions as well as to novel instances of those actions (Grossman, Blake, & Kim, 2004).

On the other hand, there is evidence that the increased motion sensitivity in adults may be forged by evolutionary pressures to rapidly detect terrestrial animals (Fox & McDaniel, 1982; Sifre et al., 2018; Simion, Regolin, & Bulf, 2008; Troje & Westhoff, 2006). Motion sensitivity to other animals is observed very early in human development. For example, developmental studies with 2-day-old human babies showed a preference to PL displays with natural motion (e.g., walking hen) relative to nonnatural motion (i.e., scrambled hen motion; Simion, Regolin, & Bulf, 2008; see also Bardi et al., 2014; Sifre et al., 2018), and this preference was specific to the upright relative to the inverted orientation. This is consistent with work showing that the inversion effect in humans, at least when making judgments of the *direction* of human and animal terrestrial movement, can be explained predominantly by the local motion of the feet (Troje & Westhoff, 2006), that is, a motion pattern not specific to humans. Again, in line with these studies using PL displays, although human targets are found more efficiently in natural scenes relative to machine targets (Mayer et al., 2015, 2017), human targets are not found more efficiently than terrestrial animal targets (Mayer et al., 2020). Thus, although perceptual learning throughout a person's individual experience can influence detection of articulatory movement, the human brain is likely to come prepackaged with circuitry that enables rapid detection of terrestrial movement.

Thus, studying human movements themselves does not clarify whether high-level, domain-specific motion sensitivity can occur as a result of naturalistic perceptual expertise, to evolutionary pressure for terrestrial motion (e.g., walking locomotion), or other factors (e.g., motor experience of performing similar actions; Casile & Giese, 2006). The current study examined if extensive *real-world* experience detecting and discriminating between natural categories influences sensitivity to natural movement of exemplars from those categories. The expert and novice birdwatchers’ task were to detect upright and inverted PL displays of flying birds and human walkers embedded in noise dots that maintained the local motion of the signal dots. The noise dots ensured that observers could not detect the stimulus based on any static frame and that the intact objects could not be recognized by form derived from the configuration of signal dots in each frame. We chose birdwatching as a domain of investigation for several reasons: (a) All birds share a highly idiosyncratic and diagnostic form of movement (i.e., synchronous movement of wings during flight), (b) experienced birdwatchers have extensive experience observing birds in flight, and (c) birds’ movement is a form of nonterrestrial movement, thereby minimizing any influence of experience with other terrestrial animals (e.g., dogs, cats, other humans). We hypothesized that extensive experience in the bird domain would increase sensitivity to the motion of flying birds. We therefore predicted that expert birdwatchers would be able to detect bird PL displays in more noise dots than novice watchers (i.e., expertise effect) and that only bird experts would show an inversion effect for bird PL displays in this detection task (i.e., enhanced sensitivity to motion patterns specific to a canonical upright condition). By comparison, since the experts and novices are likely to have equal experience with human walkers, we predicted that there would be no difference in the number of noise dots between both groups and that both groups would show the well-known inversion effect for human PL displays.

## Method

### Participants

Twenty expert participants, ranging in age from 14 to 72 years (seven females, *M* = 48.75, *SD* = 18.54), were selected based on nominations from their birdwatching peers. Twenty additional age- and education-matched participants, ranging in age from 15 to 72 years (nine females; *M* = 47.65, *SD* = 17.85), were selected to serve as the novice control group. We aimed to test as many experts as we could recruit within the 4-month period of the study and wanted to recruit at least 15 subjects based on our prior work with bird experts (Hagen et al., 2014, 2016). The novice participants were screened for having no prior experience in birdwatching. Eleven out of the 20 expert participants had taken part in previous studies on bird recognition in our lab (Hagen et al., 2014, 2016; Hagen & Tanaka, 2019). Eight of the novice participants had taken part in previous experiments.

**Figure 1.**
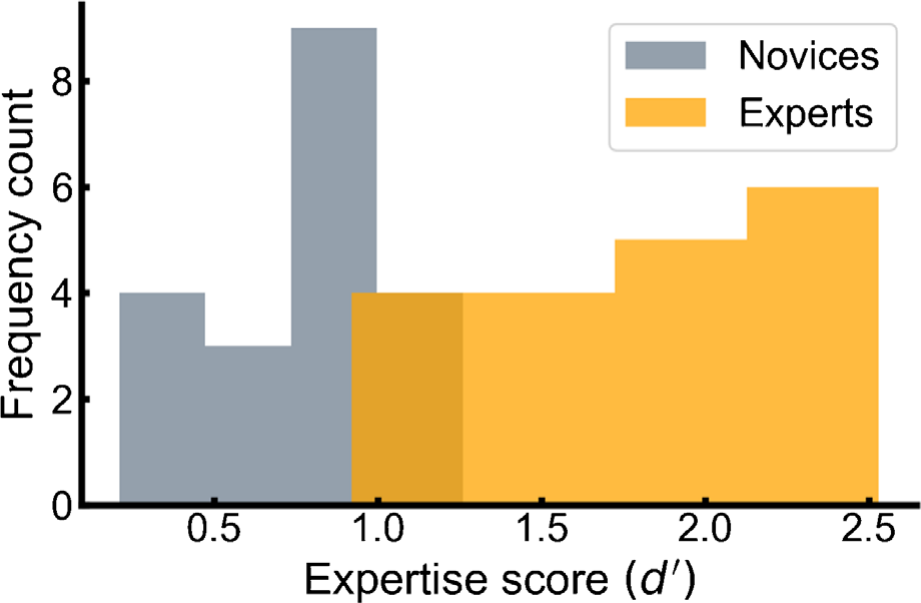
Distribution of expertise scores as a function of group (expert, novice). Dark yellow indicates overlap between the groups (*n* = 4 experts).

The criteria for expertise were based on nominations from other birdwatching peers, with the criteria that they were considered among the more capable birdwatchers and that they spent a substantial part of their spare time birdwatching in the nature. We also assessed their level of bird recognition performance with an independent bird recognition test (Hagen et al., 2014, 2016; Hagen & Tanaka, 2019), in which participants judged whether two sequentially presented bird images belonged to the same or different species. Data for one expert in the independent bird recognition test were lost due to technical issues, yielding data from 19 experts and 20 novices (the main experiment compared 20 experts with 20 novices). The scores of the experts and the novices were compared using a Welch's two-sample *t* test due to unequal sample sizes and unequal variance. The experts obtained a reliably higher discrimination score (*d*′ = 1.84, *SE* = 0.53) compared to the novices (*d*′ = 0.78, *SE* = 0.30), *t*(28.13) = 7.68 *p* < 0.001; Figure 1). While the analysis reported below included all the experts and the novices, the same analysis was run on a subset of the participants that excluded four experts and their age- and gender-matched controls due to the experts scoring lower than the best-performing novice (*n* removed = 8). These analyses yielded the same pattern as the overall analysis and are reported in the Supplementary Analysis (Figure S1).

### Design

Expert and novice participants were tested in two blocks. The PL stimuli were blocked by object domain (human, bird), and participants were told before each block which type would be presented on that block. The block order was counterbalanced across participants. Within each block, we manipulated object orientation (upright, inverted). The participants’ task was to discriminate between intact and scrambled PL displays embedded in noise dots on every trial; thus, on half the trials, the PL stimulus was scrambled (see procedure below).

### Stimuli

**Figure 2.**
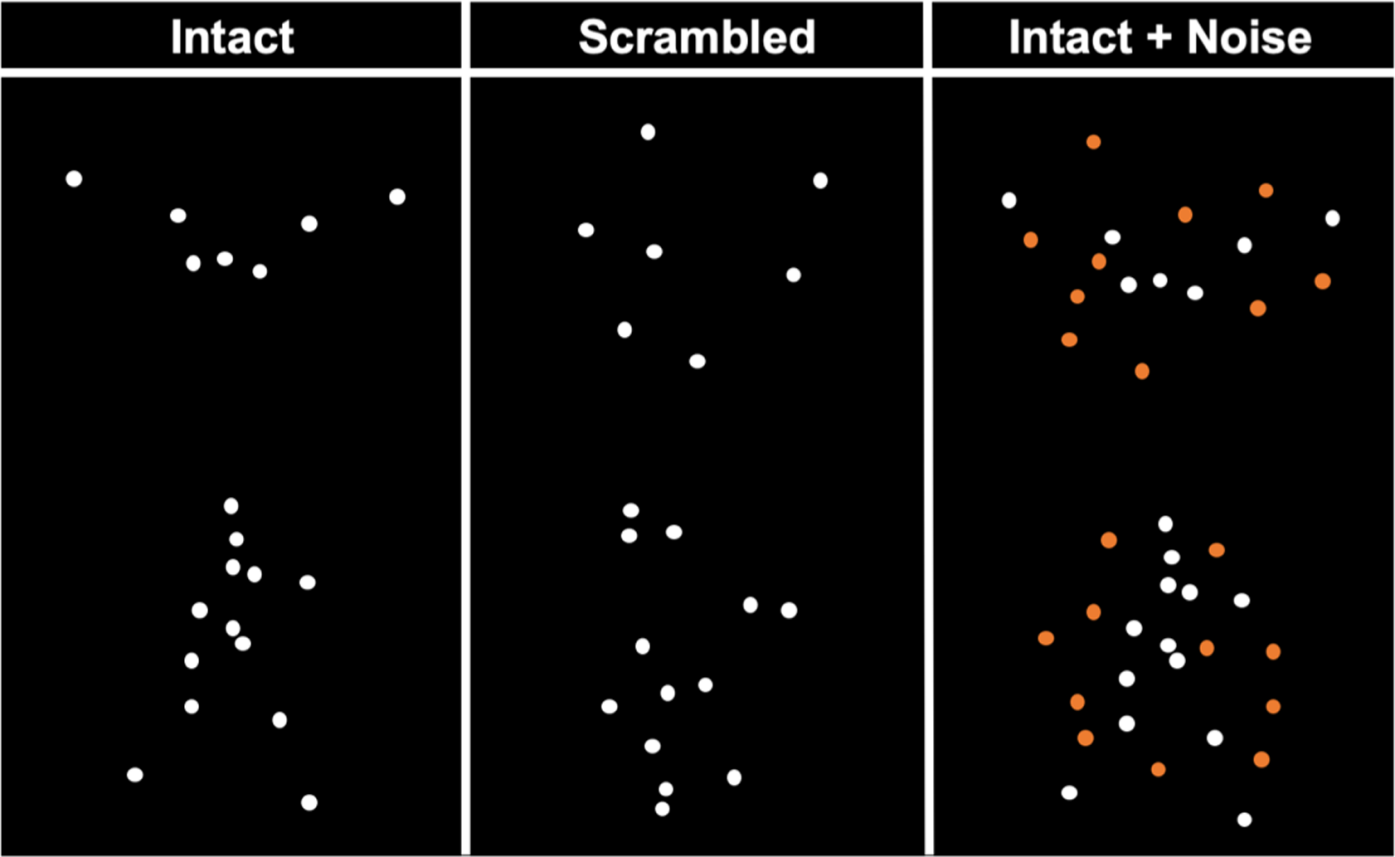
Schematic of stimuli. Left: Examples of upright point-light stimuli of intact bird (top) and intact human (bottom). Middle: Examples of point-light stimuli of scrambled bird (top) and scrambled human (bottom). Right: Examples of point-light stimuli of intact bird + noise (top) and intact human + noise (bottom). Note that orange dots are used here to illustrate the noise dots, but all dots were white in the experiment. Moreover, the participants always saw the intact/scrambled + noise condition (third column).

Bird PL displays were created from videos displaying a single bird in flight. Segments from seven different videos, each displaying a different bird in flight, which were all common to the region and known to each expert (crane, crow, duck, eagle, heron, seagull, swan), were selected to serve as the stimuli (separately, in a seven-alternative forced-choice task, a subset of participants recognized the birds in the real videos with high accuracy [experts = 96.7%, *n* = 15; novices = 78.6%, *n* = 7; chance = 14.3%]). Using a customized script in MATLAB, seven strategic points of the birds (beak [1], body [2], wings [4]) were manually tagged on each of the 75 subsequent frames (Figure 2, left column, top row; Supplementary Movie S1). The x,y-coordinates of the tags were stored for each frame and used as position coordinates for the signal dots in the PL displays. The bird PL stimuli were scaled to approximately the same width and height and did not contain any frames in which signal dots overlapped. Each presentation of a bird in flight was always centered on the screen and played for 2.5 s (75 frames at 30 Hz). In contrast, PL displays of walking humans consisted of 13 signal dots placed on strategic body positions (Figure 2, left column, bottom row, Supplementary Movie S2). These stimuli were selected from an existing three-dimensional stimulus set (Dekeyser, Verfaillie, & Vanrie, 2002; Vanrie & Verfaillie, 2004). Seven different profile views of the same walker, ranging from 150° to 210° (10° intervals), were selected to serve as stimuli. Although rotating the profile views is not equivalent to using seven different bird species, this manipulation added variability across different trials of the human walker. Each presentation of a human walker was always centered on the screen and played for 1 s (30 frames presented at 30 Hz).

Inverted human and bird PL stimuli were created by rotating the upright PL stimuli 180° in the picture plane (Supplementary Movies S3–S4). The scrambled counterparts to the intact PL stimuli were created by randomly shifting the starting position of the original signal dots. Crucially, this spatial scrambling manipulation ensured that the local motion of each dot was preserved across intact and scrambled stimuli, while disrupting the global percept of the object in motion. The scrambling procedure was carried out on a trial-by-trial basis as follows. We first defined a bounding rectangle for each PL stimulus based on its maximum height and width and divided this rectangle into nine equally-sized segments (i.e., 3 × 3 grid). We then randomly repositioned the starting position of the signal dots (i.e., the x,y-coordinates on Frame 1) within the bounding rectangle with the constraints that starting positions were randomly and equally distributed into one of the nine segments and that none of the new positions extended beyond the rectangle by more than 25 pixels (Figure 2, middle column; Supplementary Movies S5–S8). The scrambled dots and noise dots, in which the intact and scrambled PL stimuli were embedded, were generated using the same procedure. We created the desired number of noise dots for a given trial by randomly selecting signal dots with replacement and then using the same scrambling procedure (Figure 2, right column; Supplementary Movies S9–S16; note that orange dots are used here to illustrate the noise dots, but all dots were white in the experiment. The participants always saw the intact/scrambled + noise condition [third column]). The noise dots ensured that the intact objects could not be recognized by form derived from the configuration of signal dots in each frame (compare left and right column of Figure 2).

The signal dot subtended 0.10° of visual angle. Human displays subtended approximately 5.5° vertically and 2.5° horizontally, while birds subtended approximately 2.1° vertically and 4.8° horizontally. It is worth noting that human and bird PL displays differed with respect to their number of signal dots and presentation duration. Although this difference may confound the direct contrast between the object domains, they do not invalidate our goal of comparing differences between experts and novices across the two domains. The PL bird stimuli and MATLAB scripts are available upon request for noncommercial research purposes (also see Supplemental Material for example stimuli).

### Procedure

**Figure 3.**
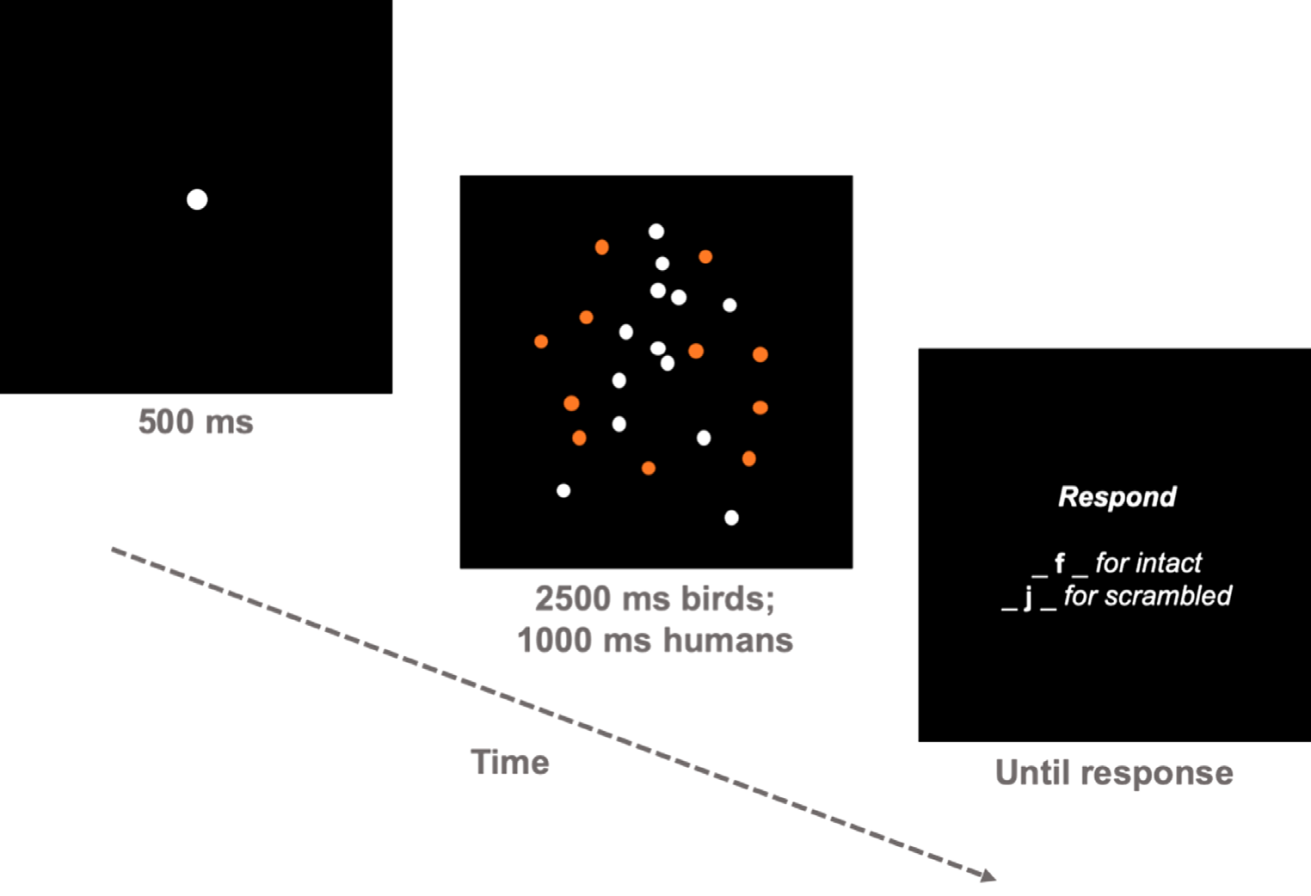
Schematic depicting the layout of a single trial. Participants saw a fixation dot for 500 ms, followed by the PL displays, which were shown for a fixed time of 2,500 ms for birds and 1,000 ms for humans. Note that the orange PL dots are used here only as illustration for highlighting the noise dots. In the actual experiment, all dots were white. After the PL displays, the participant made a key response.

On each trial, participants saw a white fixation dot presented at the center of the screen for 500 ms, followed by a PL stimulus embedded in noise and lastly followed by a response screen (Figure 3). They were instructed to press “f” on the keyboard if an intact PL stimulus was present and to press “j” if a scrambled PL stimulus was present (i.e., intact PL stimulus absent). They could only respond during the response screen to ensure that participants viewed the PL stimulus of each object domain for their full duration. Participants were informed that they would see intact and scrambled PLs of upright and inverted flying birds or walking humans embedded in noise and that the number of noise dots would vary randomly on each trial. The order of trials was randomized such that each trial had an equal probability of displaying any combination of scrambled/intact and upright/inverted PL stimuli. Thus, the participants were not able to strategize regarding the inversion condition. All the PL stimuli also had an equal probability of facing left or right.

Before starting each block, the participants performed 28 practice trials in which the PL stimuli were presented without added noise dots. Next, they performed 28 practice trials in which the PL stimuli were presented in a constant number of noise dots (*n* = 8) on each of these practice trials. The stimuli used during these practice trials were different exemplars from those used on subsequent test trials.

For the experimental blocks, we used an adaptive staircase procedure to measure participants’ sensitivity to human and bird motion patterns, defined as the number of noise dots to achieve ∼79% detection accuracy (Levitt, 1971). We systematically varied the number of noise dots in which the PL stimuli were embedded using a 3-up/1-down staircase procedure (Levitt, 1971) on each block. Three correct responses in a row within a condition (e.g., upright humans) increased the number of noise dots in the next trial for that condition (made the task more difficult), while one incorrect response decreased the number of noise dots on the next trial for that condition (made the task easier). Over successive trials, this 3-to-1 ratio converged on an accuracy of about 79% for each participant, allowing us to assess differences in sensitivity to motion patterns as a function of object domain and object orientation when accuracy is held constant. Two staircases were initiated for each orientation condition (e.g., two independent staircases for upright humans) in order to minimize any bias created by random factors in each staircase. Thus, within a block, there were four interleaved staircases operating independently (two for upright and two for inverted PL stimuli). Each staircase self-terminated after 12 reversals, excluding 4 initial reversals. Noise dots would initially increase or decrease by three dots, which changed to two dots after the fourth reversal. The first trial for each staircase would start with a random number of dots between 5 and 10 for birds and a random number between 10 and 15 for humans. The difference in the number of noise dots on Trial 1 between birds and humans was based on pilot tests showing that nonexpert participants were more sensitive to humans than birds.

The sensitivity for each participant and each condition was computed by averaging the number of noise dots in the last 10 trials for each staircase, before averaging across the two staircases in each condition.

## Results

**Figure 4.**
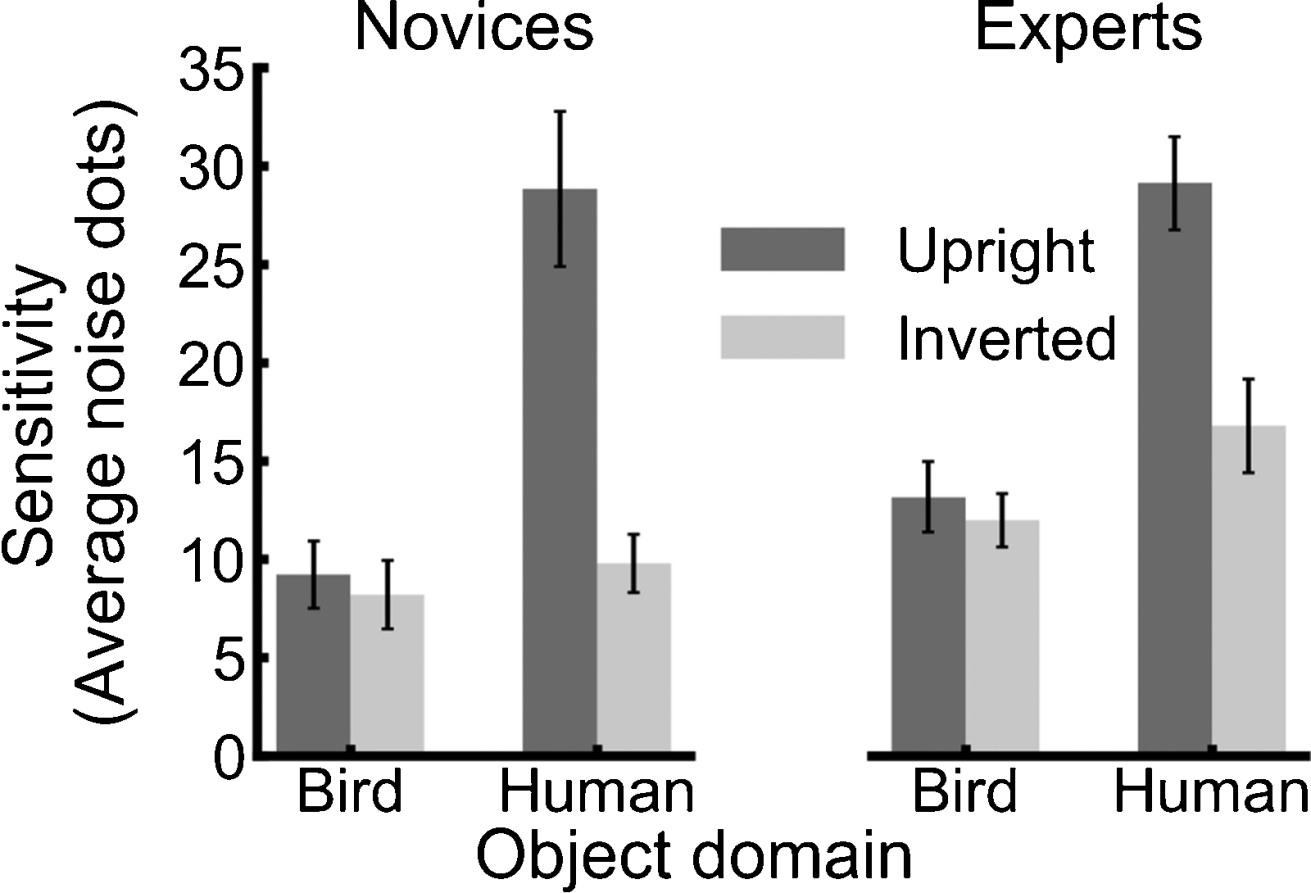
Sensitivity to motion patterns for each group (expert, novice) as a function of object domain (bird, human) and object orientation (upright, inverted). Error bars represent the *SEM*s.

Figure 4 presents mean sensitivity (i.e., average number of noise dots for ∼79% accuracy) as a function of group (novice, expert), object domain (bird, human), and object orientation (upright, inverted). The sensitivity data were analyzed in a 2 × 2 × 2 mixed-design analysis of variance (ANOVA) with group as a between-subjects factor and object domain and orientation as within-subjects factors. The main effect of group was not significant, *F*(1, 38) = 2.64, *p* = 0.112. Furthermore, group did not interact with either object domain, *F*(1, 38) = 0.006, *p* = 0.941, or object orientation, *F*(1, 38) = 1.84, *p* = 0.183. Crucially, the three-way interaction between group, object domain, and object orientation was not significant, *F*(1, 38) = 2.17, *p* = 0.149. Thus, expertise did not modulate the effects of the other factors on motion sensitivity. The same pattern was found when removing four experts with an expertise score lower than the highest-scoring novice and their age-matched controls (see Supplementary Analysis).

There were main effects and interactions for object domain and object orientation. The significant main effect of object domain, *F*(1, 38) = 59.10, *p* < 0.001, generalized eta^2^ = 0.22, showed that sensitivity was higher to humans (*M* = 21.12 dots, *SE* = 3.61 dots) than birds (*M* = 10.64 dots, *SE* = 1.89 dots). The main effect of object orientation was significant, *F*(1, 38) = 49.30, *p* < 0.001, generalized eta^2^ = 0.16. Sensitivity was higher for upright (*M* = 20.08 dots, *SE* = 3.65 dots) than inverted (*M* = 11.68 dots, *SE* = 2.12 dots) PL stimuli. These main effects were qualified by a significant interaction between object domain and object orientation, *F*(1, 38) = 38.82, *p* < 0.001, generalized eta^2^ = 0.12. Sensitivity was higher to upright humans (*M* = 28.97 dots, *SE* = 3.58 dots) than inverted humans (*M* = 13.28 dots, *SE* = 2.37 dots, *t*(39) = 6.92, *p* < 0.001). However, sensitivity did not significantly differ between upright birds (*M* = 11.19 dots, *SE* = 2.01 dots) and inverted birds (*M* = 10.08 dots, *SE* = 1.79 dots**,** *t*(39) = 1.42, *p* = 0.163).

### Bayes factor analysis

Given the null findings with respect to expertise, we conducted Bayes factor analysis to examine if the evidence favored the hypothesis that sensitivity was equal across experts and novices. We used a mixed-design repeated-measures Bayes analysis (2 group × 2 object domain × 2 object orientation) implemented in the BayesFactor package in the R programming language (Rouder, Morey, Verhagen, Swagman, & Wagenmakers, 2017) with its default settings for priors (models, priors, and methods of computation are provided in Rouder et al. [2012]). We followed the interpretation of Bayes factors of Jeffreys (1961) as adopted by Wagenmakers, Wetzels, Borsboom, and van der Maas (2011) and accepted Bayes factors > 3 as an indication for differences in performance, Bayes factors < 1/3 as an indication for consistent performance, and Bayes factors between 1/3 and 3 as anecdotal evidence for differences in performance.

The Bayes analysis was in line with the ANOVA. There was no evidence (i.e., consistent performance) for an interaction between group and object domain (Bayes factor = 0.23). Furthermore, for the bird experts, there was no evidence for a bird inversion effect (Bayes factor = 0.32). The Bayes analysis did show anecdotal evidence for group (Bayes factor = 0.85), interaction between group and object orientation (Bayes factor = 0.39), and the interaction between group, object domain, and object orientation (Bayes factor = 0.55).

In summary, there was little to no evidence for experts showing experience-dependent sensitivity to bird motion. Indeed, as shown in Table 1, while experts showed a trend toward higher bird sensitivity, this was also seen for human motion, for which experts and novices should have equal experience.

**Table 1.** Mean sensitivity scores and standard error of the mean for each group (expert, novice) and object domain (bird, human) collapsed across orientation (upright, inverted).

	Experts		Novices	
Variable	*M*	*SE*	*M*	*SE*
Bird	12.6	1.76	8.7	1.78
Human	23.0	2.89	19.3	3.6

## Discussion

The current study examined whether expertise gained from extensive experience watching birds during flight in their natural environment increased sensitivity to natural bird motion. The main findings were that the expert birdwatchers were not more sensitive to the bird flight motion than bird novices and that the experts did not show a reduction in sensitivity when flying birds were presented in an inverted orientation (Figure 4). Both groups were equally sensitive to walking humans presented in an upright orientation, and both showed a substantial decrease in sensitivity when walking humans were presented in an inverted orientation. The Bayes factor analysis further suggests that there is anecdotal or no evidence for expertise influencing detection performance. Thus, we did not find evidence to support our hypothesis that extensive real-world experience watching birds in flight would enhance sensitivity to category-specific motion (i.e., flying motion). Moreover, the inversion effect often found for humans and other terrestrial animals was not observed for birds.

Our findings corroborate previous studies showing that human observers are sensitive to PL displays of human and other animal motion (e.g., Bellefeuille & Faubert, 1998; Hiris, Humphrey, & Stout, 2005; Johansson 1973; Kaiser, Shiffrar, & Pelphrey, 2012; Mather & West, 1993; Pinto & Shiffrar, 2009; Troje & Westhoff, 2006). For example, studies have repeatedly shown that human observers can detect human walkers embedded in substantial noise and that this sensitivity is specific to the upright orientation (e.g., Cutting et al., 1988; Grossman, Blake, & Kim, 2004; Hiris, Humphrey, & Stout, 2005; Ikeda, Blake, & Watanabe, 2005; Pavlova & Sokolov, 2000; Sumi, 1984). The enhanced sensitivity to upright walkers is likely due to our lifetime worth of experience perceiving human actions in the real world. We therefore hypothesized that extensive experience perceiving birds in flight would lead to increased sensitivity to bird flight motion. We focused on the bird domain and tested whether bird experts were more sensitive than novices to articulatory bird motion in the absence of other visual cues (e.g., color or shape). If naturalistic perceptual experience influences sensitivity to domain-specific motion, then bird movement provides a strong test of this hypothesis, since it is a form of nonterrestrial movement where our perceptual experience with other terrestrial animals (e.g., dogs, cats, other humans) is unlikely to intervene. However, we found that bird experts were not disproportionately more sensitive than novices to the motion cues of birds for purposes of detection. Thus, we did not find evidence that extensive naturalistic experience observing flying birds changed perceptual sensitivity to bird flight motion. There was anecdotal evidence from the Bayes factor analysis to suggest a trend toward a higher bird sensitivity in experts. However, the experts also showed a higher sensitivity for the human domain (Table 1), suggesting that the trend was driven by nonexperiential factors (e.g., motivation). Future experiments with larger sample sizes or different domains are therefore warranted to further investigate how experts utilize different visual features or combination of visual features to recognize exemplars from their domain of expertise*.*

Although both groups showed a strong inversion effect for human motion, neither showed a bird inversion effect despite the fact that an inversion effect has been found across a range of other natural animate categories (e.g., Pinto & Shiffrar, 2009). Past research has showed that category learning, at least with static images, typically enhances sensitivity to the experienced orientation (Bukach, Gauthier, & Tarr, 2006; Gauthier & Tarr, 1997), even in the mature visual system for the already highly experienced category of faces (Laguesse et al., 2012). An inversion effect is typically interpreted as tuning of perceptual processes such that they become more sensitive with experience to larger patterns of features per eye fixation (e.g., Van Belle, De Graef, Verfaillie, Rossion, & Lefèvre, 2010). Extending this to the current findings, the inversion effect for humans could be due to sensitivity to a broader range of features in the upright than inverted orientation, similar to what is argued for images of bodies (Reed, Stone, Bozova, & Tanaka, 2003). Thus, one interpretation of the lack of inversion effect for birds in both experts and novices is that an orientation-tuned perceptual process does not develop for bird motion, perhaps because there may be no canonical “upright” orientation for aerial motion. That is, people may experience flying birds in many different orientations, in contrast to walking humans, who are always observed in the upright orientation.

Alternatively, there may exist a specialized process for the movements of human and other terrestrial animals that depend less on individual experience and rather more on evolutionary pressures to detect, for example, an approaching terrestrial threat (Fox & McDaniel, 1982; Simion, Regolin, & Bulf, 2008; Troje & Westhoff, 2006; Vallortigara, Regolin, & Marconato, 2005). This probes the exciting question as to whether specialized processes for terrestrial movement are more receptive to real-world experience than nonspecialized processes. For example, Diamond and Carey (1986) have shown that expert dog judges show an inversion effect for static dog images. It would be insightful to test whether these experts would show an inversion effect for PL dog motion. In that regard, it is worth noting that previous studies showing inversion effects for birds—even in newborns as young as 2 days old—used terrestrial movements of birds (i.e., walking hens or pigeons; Simion et al., 2008; Troje & Westhoff, 2006). Such terrestrial motion does have a natural upright orientation relative to gravity. Furthermore, the walking motion of birds still conveys some of the articulatory motion of humans (e.g., the pendular sway of two feet on the ground). Some recent studies have shown that brain regions process bipeds differently than quadrupeds, rather than natural categories per se (Papeo et al., 2017). Thus, there may be no inversion effect for flying birds because it does not activate those neural processes.

Yet, it could be that inversion disrupts domain-specific motion for which the salient movement is asymmetrically distributed and/or is moving in the orthogonal direction to the rotated axis (i.e., movement along the x-axis). Bird flight motion is symmetrically distributed across the object (i.e., the entire wing span) and moves parallel to the rotation axis (y-axis), whereas human motion is asymmetrically distributed in the lower region, is displayed in varying depth orientations (150°–210°), and moves mainly in the direction orthogonal to the rotation axis. Indeed, post hoc analysis of the PL stimuli showed that inversion not only displaced the human points on average about twice the distance of birds (human/bird = 149.13 pixels/79.58 pixels = 1.87), but human PLs also had, on average, a larger magnitude of motion along the x-axis (x = 5.16 pixels; y = 1.92 pixels) while bird PLs had a larger magnitude of motion along the y-axis (x = 2.92 pixels; y = 4.8 pixels). Thus, inversion produced a change in absolute spatial location of salient human motion while the absolute spatial position of salient bird motion remained relatively constant. Future work should use the bird flight motion to test if domain-specific motion sensitivity varies as a function of whether the pattern is presented parallel versus orthogonal to the canonical movement pattern, since a 90° rotation would result in an orthogonal shift for the birds.

While the current study did not find an expertise effect for detecting bird flight motion, it does not preclude a motion effect in expert bird identification under different circumstances or for other object domains. First, bird motion might play a larger role in recognition of birds at more specific category levels (e.g., sparrow or field sparrow) where shape and contour cues are less salient (Rosch et al., 1976). For example, expert subordinate category-level bird recognition is facilitated by domain-specific color (Hagen et al., 2014; see also Devillez, Mollison, Hagen, Tanaka, Scott & Curran, 2019) and shape (Hagen et al., 2016) information. Second, different kinds of bird motion could play a role (e.g., takeoff and landing) or depend on a specific context (e.g., flight close to ground vs. flight higher in the sky). Third, it is possible that motion sensitivity would develop for other articulatory motion if it plays a more substantial role toward the goal of the observer. For example, birdwatchers explicitly train at species-level categorization (e.g., field sparrow vs. song sparrow), where motion information is arguably less useful than shapes and color. Fourth, there may also be limitations in creating PL videos of bird flight (e.g., occlusions). Future work can also use natural videos as used for human, terrestrial-animal, and mechanical motion (Mayer et al., 2015, 2017, 2020). Thus, while the current study suggest that naturalistic exposure is not *necessarily* sufficient for developing domain-specific motion sensitivity, future work will need to further address under what circumstances it does or does not develop.

In summary, the current study sheds novel light on the role of real-world experience on enhancing sensitivity to motion of natural categories. The lack of expertise effects for aerial motion of birds suggests that sensitivity is either already at peak sensitivity—through everyday experience with such objects—or is not receptive to experiential factors in adult life. However, more work is needed to ensure that the null result generalizes to other object domains, both terrestrial and nonterrestrial, and to other paradigms and transformations of experience and canonical orientations. Nevertheless, taken together with our previous work with birdwatchers, the current study provides evidence that real-world experience with birds can lead to enhanced sensitivity to some domain-specific visual cues (e.g., color or spatial frequencies; Hagen et al., 2014, 2016) but not others (e.g., motion, this study).

## Supplementary Material

Supplement 1

Supplement 2

Supplement 3

Supplement 4

## References

[bib1] Bardi, L., Regolin, L., & Simion, F. (2014). The first time ever I saw your feet: Inversion effect in newborns’ sensitivity to biological motion. *Developmental Psychology,* 50(4), 986. 24099548 10.1037/a0034678

[bib2] Bassili, J. N. (1978). Facial motion in the perception of faces and of emotional expression. *Journal of Experimental Psychology: Human Perception and Performance,* 4(3), 373. 681886 10.1037//0096-1523.4.3.373

[bib3] Bellefeuille, A., & Faubert, J. (1998). Independence of contour and biological-motion cues for motion-defined animal shapes. *Perception,* 27(2), 225–235. 9709454 10.1068/p270225

[bib4] Bukach, C. M., Gauthier, I., & Tarr, M. J. (2006). Beyond faces and modularity: the power of an expertise framework. *Trends in Cognitive Sciences,* 10(4), 159–166. 16516534 10.1016/j.tics.2006.02.004

[bib5] Casile, A., & Giese, M. A. (2006). Nonvisual motor training influences biological motion perception. *Current Biology,* 16(1), 69–74. 16401424 10.1016/j.cub.2005.10.071

[bib6] Cavanagh, P., Labianca, A. T., & Thornton, I. M. (2001). Attention-based visual routines. Sprites. *Cognition,* 80, 47–60. 11245839 10.1016/s0010-0277(00)00153-0

[bib7] Cutting, J. E., & Kozlowski, L. T. (1977). Recognizing friends by their walk: Gait perception without familiarity cues. *Bulletin of the Psychonomic Society,* 9(5), 353–356.

[bib8] Cutting, J. E., Moore, C., & Morrison, R. (1988). Masking the motions of human gait. *Perception & Psychophysics,* 44(4), 339–347. 3226881 10.3758/bf03210415

[bib9] Dekeyser, M., Verfaillie, K., & Vanrie, J. (2002). Creating stimuli for the study of biological-motion perception. *Behavior Research Methods Instruments, & Computers**,* 34, 375–382. 10.3758/bf03195465 12395553

[bib10] Devillez, H., Mollison, M. V., Hagen, S., Tanaka, J. W., Scott, L. S., & Curran, T. (2019). Color and spatial frequency differentially impact early stages of perceptual expertise training. *Neuropsychologia,* 122, 62–75. 30471254 10.1016/j.neuropsychologia.2018.11.011

[bib11] Diamond, R., & Carey, S. (1986). Why faces are and are not special: An effect of expertise. *Journal of Experimental Psychology: General,* 115(2), 107. 2940312 10.1037//0096-3445.115.2.107

[bib12] Dittrich, W. H. (1993). Action categories and the perception of biological motion. *Perception,* 22(1), 15–22. 8474831 10.1068/p220015

[bib13] Dittrich, W. H., Troscianko, T., Lea, S. E., & Morgan, D. (1996). Perception of emotion from dynamic point-light displays represented in dance. *Perception,* 25(6), 727–738. 8888304 10.1068/p250727

[bib14] Fox, R., & McDaniel, C. (1982). The perception of biological motion by human infants. *Science,* 218(4571), 486–487. 7123249 10.1126/science.7123249

[bib15] Gauthier, I., & Tarr, M. J. (1997). Becoming a “Greeble” expert: Exploring mechanisms for face recognition. *Vision Research,* 37(12), 1673–1682. 9231232 10.1016/s0042-6989(96)00286-6

[bib17] Grossman, E. D., Blake, R., & Kim, C. Y. (2004). Learning to see biological motion: Brain activity parallels behavior. *Journal of Cognitive Neuroscience,* 16(9), 1669–1679. 15601527 10.1162/0898929042568569

[bib18] Grossman, E. D., Blake, R., & Kim, C. Y. (2006). Learning to see biological motion: brain activity parallels behavior. *Learning,* 16(9). 10.1162/0898929042568569 15601527

[bib21] Hagen, S., & Tanaka, J. W. (2019). Examining the neural correlates of within-category discrimination in face and non-face expert recognition. *Neuropsychologia,* 124, 44–54. 30659863 10.1016/j.neuropsychologia.2019.01.005

[bib19] Hagen, S., Vuong, Q. C., Scott, L. S., Curran, T., & Tanaka, J. W. (2014). The role of color in expert object recognition. *Journal of Vision*, 14(9), 9, 10.1167/14.9.9. 25113021

[bib20] Hagen, S., Vuong, Q. C., Scott, L. S., Curran, T., & Tanaka, J. W. (2016). The role of spatial frequency in expert object recognition. *Journal of Experimental Psychology: Human Perception and Performance,* 42(3), 413. 26480250 10.1037/xhp0000139

[bib22] Hiris, E., Humphrey, D., & Stout, A. (2005). Temporal properties in masking biological motion. *Perception & Psychophysics,* 67(3), 435–443. 16119392 10.3758/bf03193322

[bib23] Ikeda, H., Blake, R., & Watanabe, K. (2005). Eccentric perception of biological motion is unscalably poor. *Vision Research,* 45(15), 1935–1943. 15820512 10.1016/j.visres.2005.02.001

[bib24] Jastorff, J., Kourtzi, Z., & Giese, M. A. (2002). Learning of the discrimination of artificial complex biological motion. *Dynamic Perception,* 133–138.

[bib25] Jastorff, J., Kourtzi, Z., & Giese, M. A. (2006). Learning to discriminate complex movements: Biological versus artificial trajectories. *Journal of Vision*, 6(8), 3, 10.1167/6.8.3. 16895459

[bib26] Jeffreys, H. (1961). *Theory of probability**.* Oxford, UK: Oxford University Press.

[bib27] Johansson, G. (1973). Visual perception of biological motion and a model for its analysis. *Perception & Psychophysics,* 14(2), 201–211.

[bib28] Johnson, K., & Shiffrar, M. (2013). *People watching: Social, perceptual, and neurophysiological studies of body perception*. Oxford, UK: Oxford University Press.

[bib29] Kaiser, M. D., Shiffrar, M., & Pelphrey, K. A. (2012). Socially tuned: Brain responses differentiating human and animal motion. *Social Neuroscience,* 7, 301–310. 21943047 10.1080/17470919.2011.614003

[bib30] Kozlowski, L. T., & Cutting, J. E. (1977). Recognizing the sex of a walker from a dynamic point-light display. *Attention, Perception, & Psychophysics,* 21(6), 575–580.

[bib31] Laguesse, R., Dormal, G., Biervoye, A., Kuefner, D., & Rossion, B. (2012). Extensive visual training in adulthood significantly reduces the face inversion effect. *Journal of Vision*, 12(10), 14, 10.1167/12.10.14. 23019119

[bib32] Levitt, H. (1971). Transformed up-down methods in psychoacoustics. *Journal of the Acoustical Society of America,* 49, 467–477. 5541744

[bib33] Mather, G., & West, S. (1993). Recognition of animal locomotion from dynamic point-light displays. *Perception,* 22(7), 759–766. 8115234 10.1068/p220759

[bib34] Mayer, K. M., Vuong, Q. C., & Thornton, I. M. (2015). Do people “pop out”? *PLoS ONE,* 10, e139618. 10.1371/journal.pone.0139618 PMC4595219 26441221

[bib35] Mayer, K. M., Vuong, Q. C., & Thornton, I. M. (2017). Humans are detected more efficiently than machines in the context of natural scenes. *Japanese Psychological Research,* 59, 178–187.

[bib36] Mayer, K. M., Thornton, I. M., & Vuong, Q. C. (2020). Comparable search efficiency for human and animal targets in the context of natural scenes. *Attention, Perception and Psychophysics*, 1–12. 10.3758/s13414-019-01901-6 31686377

[bib37] Papeo, L., Wurm, M. F., Oosterhof, N. N., & Caramazza, A. (2017). The neural representation of human versus nonhuman bipeds and quadrupeds. *Scientific Reports,* 7, 14040. 29070901 10.1038/s41598-017-14424-7 PMC5656636

[bib38] Pavlova, M., & Sokolov, A. (2000). Orientation specificity in biological motion perception. *Perception & Psychophysics,* 62(5), 889–899. 10997036 10.3758/bf03212075

[bib39] Pica, P., Jackson, S., Blake, R., & Troje, N. F. (2011). Comparing biological motion perception in two distinct human societies. *PLoS One,* 6(12). 10.1371/journal.pone.0028391 PMC3237441 22194831

[bib40] Pinto, J., & Shiffrar, M. (2009). The visual perception of human and animal motion in point-light displays. *Social Neuroscience,* 4, 332–346. 19340632 10.1080/17470910902826820

[bib41] Pollick, F. E., Paterson, H. M., Bruderlin, A., & Sanford, A. J. (2001). Perceiving affect from arm movement. *Cognition,* 82(2), B51–B61. 11716834 10.1016/s0010-0277(01)00147-0

[bib42] Pyles, J. A., Garcia, J. O., Hoffman, D. D., & Grossman, E. D. (2007). Visual perception and neural correlates of novel “biological motion.” *Vision Research,* 47, 2786–2797. 17825349 10.1016/j.visres.2007.07.017

[bib43] Reed, C. L., Stone, V. E., Bozova, S., & Tanaka, J. (2003). The body-inversion effect. *Psychological Science,* 14(4), 302–308. 12807401 10.1111/1467-9280.14431

[bib44] Rosch, E., Mervis, C. B., Gray, W. D., Johnson, D. M., & Boyes-Braem, P. (1976). Basic objects in natural categories. *Cognitive Psychology,* 8(3), 382–439.

[bib45] Rouder, J. N., Morey, R. D., Speckman, P. L., & Province, J. M. (2012). Default Bayes factors for ANOVA designs. *Journal of Mathematical Psychology,* 56, 356–374.

[bib46] Rouder, J. N., Morey, R. D., Verhagen, J., Swagman, A. R., & Wagenmakers, E. J. (2017). Bayesian analysis of factorial designs. *Psychological Methods,* 22(2), 304. 27280448 10.1037/met0000057

[bib47] Sifre, R., Olson, L., Gillespie, S., Klin, A., Jones, W., & Shultz, S. (2018). A longitudinal investigation of preferential attention to biological motion in 2-to 24-month-old infants. *Scientific Reports,* 8(1), 1–10. 29410484 10.1038/s41598-018-20808-0 PMC5802706

[bib48] Simion, F., Regolin, L., & Bulf, H. (2008). A predisposition for biological motion in the newborn baby. *Proceedings of the National Academy of Sciences,* 105(2), 809–813. 10.1073/pnas.0707021105 PMC2206618 18174333

[bib49] Sumi, S. (1984). Upside-down presentation of the Johansson moving light-spot pattern. *Perception,* 13(3), 283–286. 6514513 10.1068/p130283

[bib50] Tanaka, J. W., & Taylor, M. (1991). Object categories and expertise: Is the basic level in the eye of the beholder? *Cognitive Psychology,* 23(3), 457–482.

[bib51] Thornton, I. M., & Vuong, Q. C. (2004). Incidental processing of biological motion. *Current Biology,* 14, 1084–1089. 15203001 10.1016/j.cub.2004.06.025

[bib52] Troje, N. F. (2002). Decomposing biological motion: A framework for analysis and synthesis of human gait patterns. *Journal of Vision*, 2(5), 2, 10.1167/2.5.2. 12678652

[bib53] Troje, N. F., & Westhoff, C. (2006). The inversion effect in biological motion perception: Evidence for a “life detector”? *Current Biology,* 16(8), 821–824. 16631591 10.1016/j.cub.2006.03.022

[bib54] Vallortigara, G., Regolin, L., & Marconato, F. (2005). Visually inexperienced chicks exhibit spontaneous preference for biological motion patterns. *PLoS Biology,* 3(7), e208. 15934787 10.1371/journal.pbio.0030208 PMC1150290

[bib55] Van Belle, G., De Graef, P., Verfaillie, K., Rossion, B., & Lefèvre, P. (2010). Face inversion impairs holistic perception: Evidence from gaze-contingent stimulation. *Journal of Vision*, 10(5), 10–10. 10.1167/10.5.10 20616142

[bib56] Vanrie, J., & Verfaillie, K. (2004). Perception of biological motion: A stimulus set of human point-light actions. *Behavior Research Methods, Instruments, & Computers**,* 36*,* 625–629. 10.3758/bf03206542 15641407

[bib57] Wagenmakers, E.-J., Wetzels, R., Borsboom, D., & van der Maas, H. L. J. (2011). Why psychologists must change the way they analyze their data: The case of psi: Comment on Bem (2011). *Journal of Personality and Social Psychology**,* 100*,* 426–432. 21280965 10.1037/a0022790

