# Supplementary figures and images for: Bird expertise does not increase motion sensitivity to bird flight motion
Source: J Vis. 2021 May 5;21(5):5. doi: 10.1167/jov.21.5.5 (PMC8107655; doi:10.1167/jov.21.5.5)

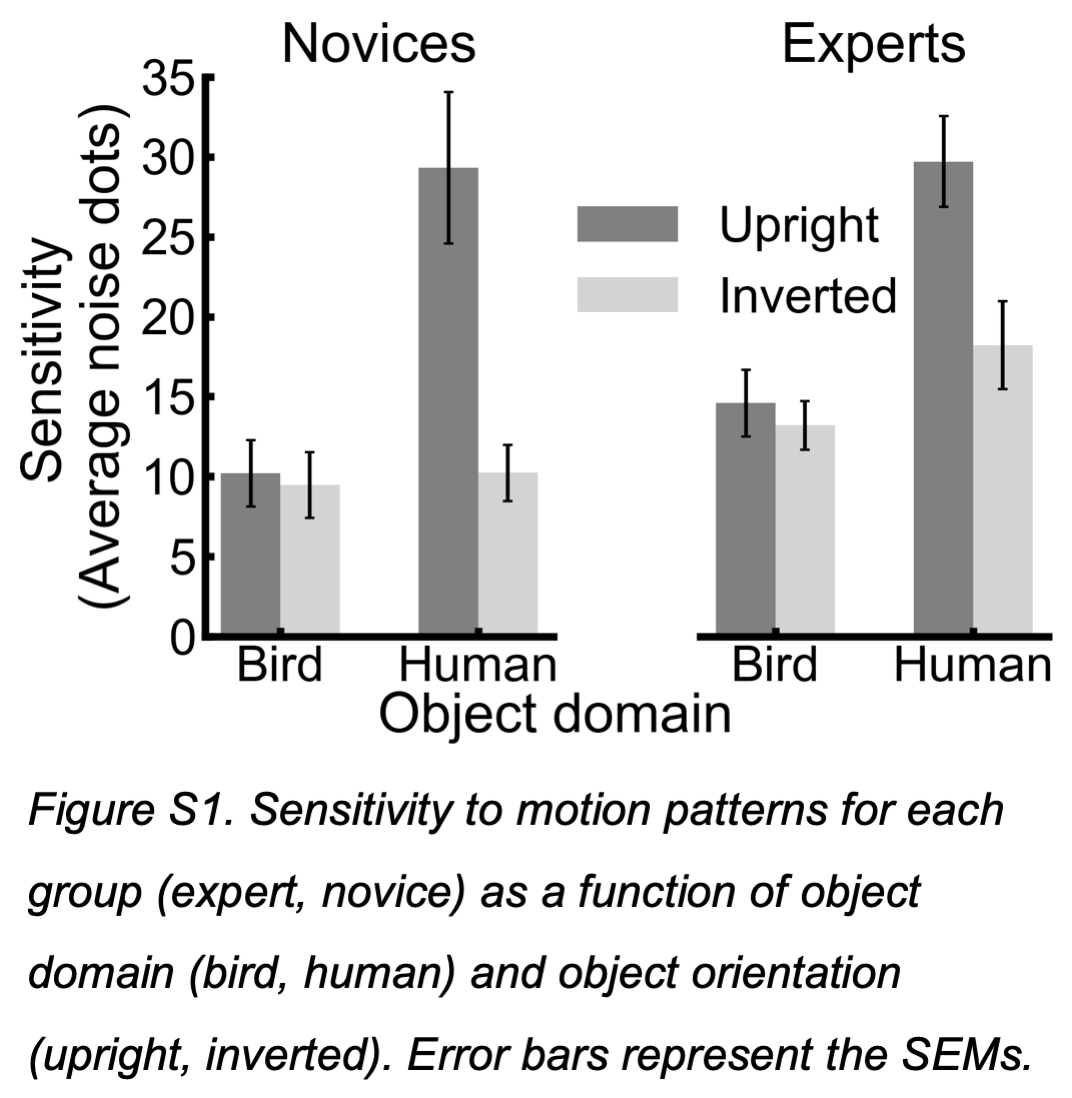

Supplement: Supplement 3 [file jovi-21-5-5_s003.png]
