# Supplementary material for: Bird expertise does not increase motion sensitivity to bird flight motion
Source: J Vis. 2021 May 5;21(5):5. doi: 10.1167/jov.21.5.5 (PMC8107655; doi:10.1167/jov.21.5.5)
Supplement: Supplement 4 [file jovi-21-5-5_s004.pdf]

Supplementary Material

Supplemental analysis

Figure S1 presents mean sensitivity scores (i.e., average number of noise dots for ~79% accuracy) as a function of group (novice, expert), object domain (bird, human) and object orientation (upright, inverted) for a subset of the participants (removed four experts and their matched controls due to low expertise score in the former). The sensitivity data were analyzed in a 2 x 2 x 2 mixed-design analysis of variance (ANOVA) with group as a between-subjects factor, and object domain and orientation as within-subjects factors. This analysis mirrored the pattern found for the main analysis with all the participants. The main effect of group was not significant, $F(1, 30) = 2.28$, $p = 0.141$. Furthermore, group did not interact with either object domain, $F(1, 30) = 0.002$, $p = 0.969$, or object orientation, $F(1, 30) = 1.44$, $p = 0.240$. Crucially, the three-way interaction between group, object domain, and object orientation was not significant, $F(1, 30) = 2.28$, $p = 0.141$. Thus, expertise did not seem to modulate the effects of the other factors on motion sensitivity.

There were main effects and interactions for object domain and object orientation. The significant main effect of object domain, $F(1, 30) = 37.51$, $p < 0.001$, generalized $eta^2 = 0.19$. The main effect of object orientation was significant, $F(1, 30) = 31.92$, $p < 0.001$, generalized $eta^2 = 0.14$. These main effects were qualified by a significant interaction between object domain and object orientation, $F(1, 30) = 27.08$, $p < 0.001$, generalized $eta^2 = 0.11$.

Bayes Factor Analysis. The Bayes analysis was in line with the ANOVA. There was no evidence (i.e., consistent performance) for an interaction between group and object domain (Bayes Factor = 0.25). Furthermore, for the bird experts, there was no evidence for a bird inversion effect (Bayes Factor = 0.35). The Bayes analysis did show anecdotal evidence for group (Bayes Factor = 0.90); interaction between group and object orientation (Bayes Factor = 0.40); and the interaction between group, object domain and object orientation (Bayes Factor = 0.65).
